# Edible algae (*Ecklonia cava*) bioprocessed with mycelia of shiitake (*Lentinula edodes*) mushrooms in liquid culture and its isolated fractions protect mice against allergic asthma

**DOI:** 10.1186/s12906-022-03705-y

**Published:** 2022-09-17

**Authors:** Kyung Hee Lee, Yeo Jin Jang, Woon Sang Hwang, Ki Sun Kwon, Wha Young Lee, Jeanman Kim, Sung Phil Kim, Mendel Friedman

**Affiliations:** 1 STR Biotech Co., Ltd., Chuncheon, Republic of Korea; 2 Present address: Herbal Medicine Research Division, National Institute of Food & Drug Safety Evaluation, Cheongju, Republic of Korea; 3 U.S. Department of Agriculture, Western Regional Research Center, Agricultural Research Services, Albany, CA USA

**Keywords:** Edible algae, *Ecklonia cava*, Shiitake mushrooms, Bioprocessed food, mast cells, Mice, Gas chromatography, Bioassays, Dietary interventions, Immunoglobulins, cytokines, Bronchoalveolar lavage fluid, Lungs, Histology, Anti-asthma effect

## Abstract

**Background:**

*Ecklonia cava* is an edible marine brown alga harvested from the ocean that is widely consumed in Asian countries as a health-promoting medicinal food The objective of the present study is to evaluate the anti-asthma mechanism of a new functional food produced by bioprocessing edible algae *Ecklonia cava* and shiitake *Lentinula edodes* mushroom mycelia and isolated fractions.

**Methods:**

We used as series of methods, including high performance liquid chromatography, gas chromatography, cell assays, and an in vivo mouse assay to evaluate the asthma-inhibitory effect of *Ecklonia cava* bioprocessed (fermented) with *Lentinula* edodes shiitake mushroom mycelium and its isolated fractions in mast cells and in orally fed mice.

**Results:**

The treatments inhibited the degranulation of RBL-2H3 cells and immunoglobulin E (IgE) production, suggesting anti-asthma effects in vitro. The in vitro anti-asthma effects in cells were confirmed in mice following the induction of asthma by alumina and chicken egg ovalbumin (OVA). Oral administration of the bioprocessed *Ecklonia cava* and purified fractions suppressed the induction of asthma and was accompanied by the inhibition of inflammation- and immune-related substances, including eotaxin; thymic stromal lymphopoietin (TSLP); OVA-specific IgE; leukotriene C_4_ (LTC4); prostaglandin D2 (PGD_2_); and vascular cell adhesion molecule-1 (VCAM-1) in bronchoalveolar lavage fluid (BALF) and other fluids and organs. Th2 cytokines were reduced and Th1 cytokines were restored in serum, suggesting the asthma-induced inhibitory effect is regulated by the balance of the Th1/Th2 immune response. Serum levels of IL-10, a regulatory T cell (Treg) cytokine, were increased, further favoring reduced inflammation. Histology of lung tissues revealed that the treatment also reversed the thickening of the airway wall and the contraction and infiltration of bronchial and blood vessels and perialveolar inflammatory cells. The bioprocessed *Ecklonia cava/mushroom* mycelia new functional food showed the highest inhibition as compared with commercial algae and the fractions isolated from the bioprocessed product.

**Conclusions:**

The in vitro cell and in vivo mouse assays demonstrate the potential value of the new bioprocessed formulation as an anti-inflammatory and anti-allergic combination of natural compounds against allergic asthma and might also ameliorate allergic manifestations of foods, drugs, and viral infections.

## Background

Asthma is a chronic human inflammatory disease of lung airways caused by triggering stimuli that include dust, food, food-derived inhalants, obesity, pollen, smoke as well as ethnicity, socioeconomic, and environmental factors causing partially or completely reversible constriction of the lung bronchi that requires ongoing medical management [1–6].

Treatment involves minimizing or eliminating triggering factors and self-administered over-the-counter and physician-prescribed drug therapy that suppresses various so-called biomarkers associated with the asthmatic syndrome. Asthma-associated biomarkers include immune cells (T-helper, Th1, Th2), cytokines (IL-4, IL-13), eosinophils, mast cells, and neutrophils that form an inflammatory infiltrate in the lung airways causing adverse symptoms.

The alga *Ecklonia cava* is an edible medicinal food that is widely consumed in Korea and Japan for its reported multiple health benefits that include anti-cancer [7], anti-diabetes [8], anti-inflammatory [9], and anti-obesity [10] properties. These studies from two publications suggest that the alga and isolated bioactive compounds might be safe for human consumption.

On the basis of the results of an investigation of acute and subchronic oral toxicity and genotoxicity of an enzymatic extract of *Ecklonia cava*, Yun, et al. [11] concluded that the extract seems safe as a potential therapeutic for human consumption against various diseases: (a) acute oral LD_50_ values of the administered to female and male rats and dogs equals > 3000 mg/kg body weight; (b) potential toxic symptoms, including food/water consumption, mortality, urinalysis, hematology, serum biochemistry, organ weight, or histopathology were not affected by the treatment; and (c) data from the Ames test mutagenicity, chromosome aberration assay, and micronucleus assay indicated that the extract was not mutagenic or clastogenic.

Because metabolomic analysis is a powerful tool for the investigation of interactions between diet, nutrients, and human metabolism, Kim, et al. [12] investigated the effect of consuming the phenolic compound seapolynol, isolated from *E. cava*, on the urinary metabolomic profile of human volunteers. The results show that levels of riboflavin, urocanic acid, 5-hydroxy-6-methoxyindole glucuronide, and guanidino valeric acid were significantly increased in the seapolynol group compared with the placebo group and that urinary riboflavin was associated with BMI, body weight, fat mass, and percent body fat, suggesting that the decreased body fat induced by the intake of seapolynol is related to an increase in the antioxidant effect of riboflavin.

In a previous study, we investigated the efficacy and mechanism of the anti-asthma effect of an *Ulmus parvifolia* bark extract bioprocessed in *Lentinula edodes* liquid mycelium culture against allergic asthma in chicken egg ovalbumin (OVA)sensitized/challenged mice [13]. The results suggested that the oral administration of the food formulation to asthmatic mice prevented allergic asthma. Because of the above cited health benefits and safety aspects, the aim of this study was to determine the anti-asthma effect and its mechanism of a bioprocessed (fermented) *Ecklonia cava* new functional food and of isolated fractions**.**

## Materials and methods

### Materials

Dulbecco’s modified Eagle’s medium (DMEM), phosphate-buffered saline (PBS), fetal bovine serum (FBS), and other miscellaneous cell culture reagents were purchased from Thermo Fisher Scientific (Waltham, MA, USA). Potato dextrose agar medium (PDA) was from Becton, Dickinson Company (Franklin Lakes, NJ, USA). Chicken egg ovalbumin (OVA, grade V), human IL-4, aluminum hydroxide, and analytical grade reagents were obtained from MERCK (Darmstadt, Germany). *Ecklonia cava* was purchased at Suchang Eco Bio Co., Ltd. (Jeju, Korea).

#### Preparation of bioprocessed *Ecklonia cava* and isolated fractions

Bioprocessing was carried out according to the method we previously reported with some modifications [13–15]. We selected the *Lentinula edodes* for this study because as reported in the cited publications, we previously used this mushroom variety to create other health-promoting bioprocessed functional foods. In addition, compared with other mushroom mycelia in previous studies, the *Lentinula edodes* mycelium showed the highest physiological activity at 28 °C.

Briefly, *Lentinula edodes* fungal mycelia were isolated from the fruit body and cultured on PDA and the genetic identity of the fungus was confirmed by the Korean Center of Microorganisms (Seoul, Korea). The mycelia were then inoculated using an inoculating loop into an Erlenmeyer flask containing 50 mL of the liquid medium (2% glucose, 0.5% yeast extract, 0.5% soy peptone, 0.2% KH_2_PO_4_, 0.05% MgSO_4_, and 0.002% FeSO_4_, w/v), and then cultured at 28 °C for 5 days in a rotary shaker (120 rpm). The main culture then seeded with this culture. Minced *Ecklonia cava* was added to DMEM (30 g/L) and treated with amylase (40 U) and cellulase (100 U) at 60 °C for 60 min for the enzymatic digestion of carbohydrates. The digest was then adjusted to pH 7.0 and sterilized in an autoclave. The *Ecklonia* preparation was added to a 5 L fermenter, inoculated with the seed culture, and cultured at 28 °C and 150 rpm for 5 days. This main culture was treated with an enzyme mixture containing glucanase (3300 U), cellulose (6000 U), hemicellulose (3000 U), and pectinase (3000 U) at 60 °C for 60 min for cell wall lysis and then heated at 90 °C for 1 h. The resultant aqueous phase was recovered and freeze-dried to a solid material. This material was termed EC-F-1 (bioprocessed *Ecklonia cava* extract). *Ecklonia cava* extract not subjected to bioprocessing was also prepared under enzymatic hydrolysis, extraction with water (90 °C/1 h) or 70% ethanol, and freeze-drying conditions, and termed EC-1 (water extract) and EC-2 (70% ethanol extract).

**Fig. 1:**
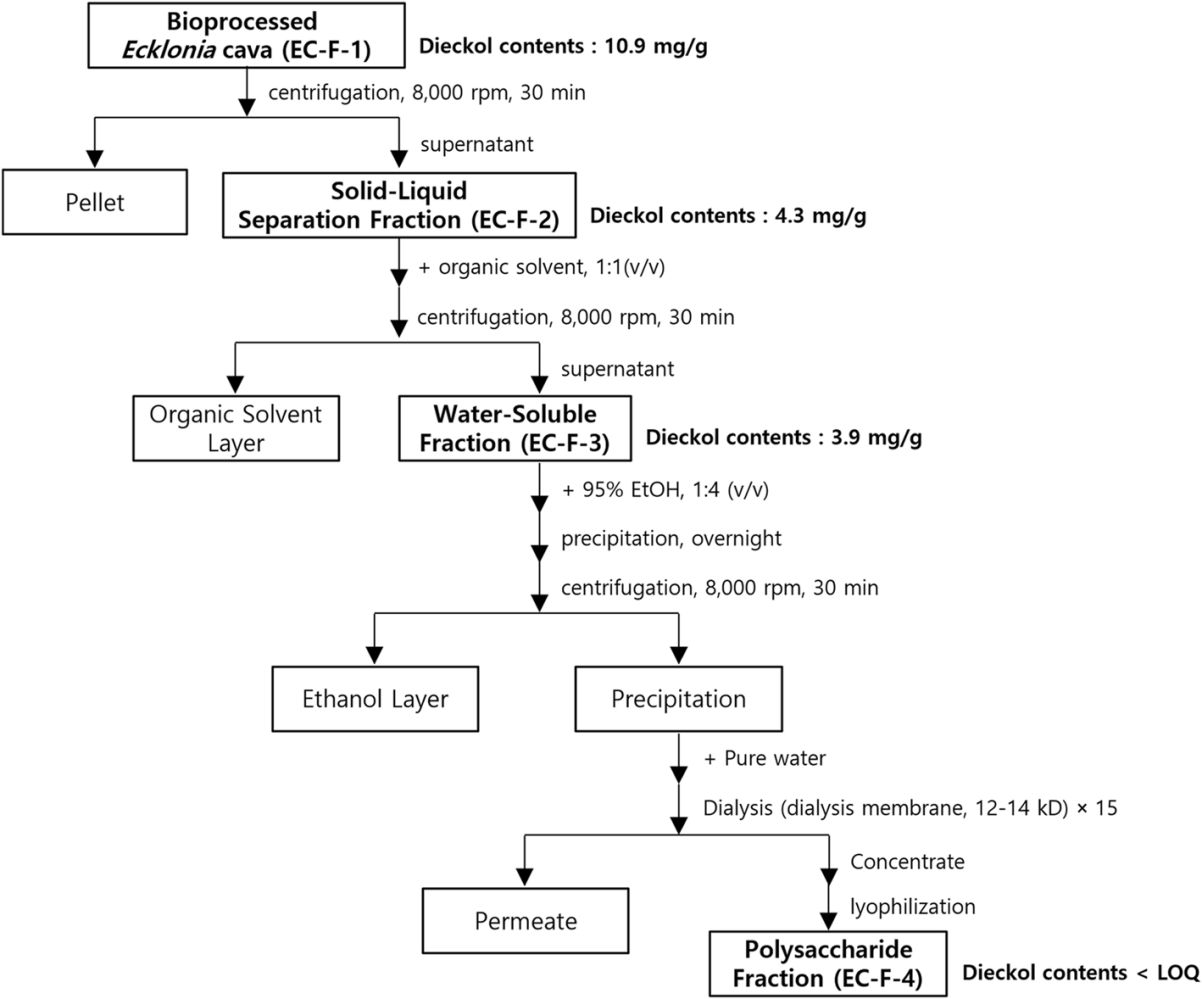
Scheme for the purification procedure of bioprocessed *Ecklonia cava*

The bioprocessed *Ecklonia cava* was then purified by a published method for isolation of polysaccharides with some modification [16]. The dried powder of bioprocessed *Ecklonia cava* (50 g) was then dissolved by stirring with pure water at room temperature for 24 h, and the insoluble residue was then removed by centrifugation (8000 rpm 30 min). The centrifugation supernatant was designated as a solid–liquid separation fraction of bioprocessed *Ecklonia cava* (EC-F-2). An organic solvent was used to remove impurities from the lipid-like component of the solid-liquid separation fraction of bioprocessed *Ecklonia cava*. The organic solvent chloroform in the same amount as the solid-liquid separation fraction, was mixed, then centrifuged to separate the organic solvent layer and the aqueous layer. At this point, all of the immune-active polysaccharide components were present in the aqueous layer. The aqueous layer was designated as a water-soluble fraction of bioprocessed *Ecklonia cava* (EC-F-3). The water-soluble fraction was then precipitated by the addition of ethanol (4 times the volume of a water-soluble fraction) at 4 °C followed by centrifugation at 8000 rpm (30 min). The resulting precipitate was dissolved in water (150 mL), then dialyzed (12-14 K Dalton) against pure water for 2 days and lyophilized to yield the polysaccharide fraction of bioprocessed *Ecklonia cava* (EC-F-4, 0.87 g). Figure 1 shows the purification procedure of bioprocessed *Ecklonia cava*.

#### HPLC analysis of dieckol from isolated fractions of bioprocessed *Ecklonia cava*

The HPLC method was adapted from the literature [17]. The analysis was performed using a reverse-phase HPLC system (Shimadzu LC-20A, Shimadzu Co. Kyoto, Japan) equipped with a binary pump, an autosampler, a column oven and a PDA detector. A Supelco Discovery C18 column (150 × 4.6 mm i.d., 5 μm, MERCK) in a column oven set at 25 °C was used for separations. The mobile phase consisted of deionized water containing 0.1% formic acid (mobile phase A) and acetonitrile containing 0.1% formic acid (mobile phase B). The gradient elution conditions were as follows: 0–17 min, 13–15% B; 17–30 min, 15–27% B; 30–40 min, 27% B; 40–45 min, 27–100% B; 45–48 min, 100% B; 48–50 min, 100–13% B; 50–60 min, 13% B at a flow rate of 1.0 mL/min. The sample injection volume (20 μL) and ultraviolet (UV) absorbance was monitored at 320 nm.

#### Gas chromatography analysis of saccharides in the isolated polysaccharide fraction of bioprocessed *Ecklonia cava*

Gas chromatography with a flame ionization detector (GC-FID) was performed by a method adapted from the literature with some modifications [18, 19]. Briefly, the polysaccharide fraction (20 mg) was dissolved in trifluoroacetic acid (14 mL, 2 M) in a test tube with a Teflon screw cap, which was then sealed in vacuum and incubated in a heating block (100 °C, 6 h). The tube was then opened and the trifluoroacetic acid was removed at 70 °C under a steady stream of nitrogen. The dried hydrolyzed polysaccharide fraction was transferred to Teflon screw cap tubes and acetyl inositol (2 mg) was added as an internal standard. The following monosaccharide standards (10 mg) were also used: ribose, rhamnose, arabinose, xylose, fucose, xylitol, mannose, glucose, galactose, sorbitol and galactosamine. This was followed by addition of pyridine (0.5 mL) and hydroxylamine hydrochloride (10 mg) to each tube. The sealed tubes were incubated in a preheated water bath shaker at 90 °C for 30 min. Acetic anhydride (0.5 mL) was then added to the cooled tubes at room temperature. The vortexed tubes were then sealed and incubated in a water bath shaker set at 90 °C for 30 min, cooled and the clear supernatant (0.1 mL), and acetic anhydride (0.5 mL) were added to autosampler vials with inserts for injection into the gas chromatograph.

The sugars we then analyzed under the following conditions. The system used was a Shimadzu GC-2010 fitted with a capillary column HP-5 (30 m × 0.32 mm i.d., film thickness 0.25 mm) and a flame ionization detector with high-purity helium as the carrier gas at a flow rate of 1.2 mL/min. The oven temperature was initially at 160 °C, held for 2 min, then ramped at the rate of 5 °C/min to 240 °C and held for 10 min. Injections were performed in the splitless mode.

#### Cell culture procedure

The transformed rat basophilic leukemia mast cell line RBL-2H3 from American Type Tissue Culture Collection (ATCC, Manassas, VA, USA) was cultured in a modified DMEM containing 10 mM HEPES, 2 mM L-glutamine, 1 mM sodium pyruvate, 4.5 g/L glucose, 1.5 g/L sodium bicarbonate, and 10% heat-inactivated FBS with added penicillin (100 U/mL) and streptomycin (100 mg/mL). Cells were cultured at 37 °C in a humidified atmosphere with 5% CO_2_. The U266.B1 human multiple myeloma B lymphocyte cells from American Type Tissue Culture Collection (ATCC, Manassas, VA, USA) were cultured in a modified RPMI1640 medium containing 10 mM HEPES, 2 mM L-glutamine, 1 mM sodium pyruvate, 4.5 g/L glucose, 1.5 g/L sodium bicarbonate, and 15% heat-inactivated FBS with added penicillin (100 U/mL) and streptomycin (100 mg/mL). Cells were cultured at 37 °C in a humidified atmosphere with 5% CO_2_.

#### β-Hexosaminidase release assay

As an indicator of an allergic reaction, β-hexosaminidase secretion in the RBL-2H3 cell line was determined using a published method with some modifications [20]. Briefly, RBL-2H3 cells were cultured in a 96-well plate (1 × 10^5^ cells/mL). The extracts (EC-1, *Ecklonia cava* water extracts (1, 10, 100 μg/mL); EC-2, *Ecklonia cava* 70% ethanol extracts (0.41, 4.1, 41 μg/mL); EC-F-1, bioprocessed *Ecklonia cava* extract (1, 10, 100 μg/mL); EC-F-2, solid-liquid separation fraction of bioprocessed *Ecklonia cava* extracts (0.31, 3.1, 31 μg/mL); EC-F-3, water-soluble fraction of bioprocessed *Ecklonia cava* extracts (0.29, 2.9, 29 μg/mL); EC-F-4, polysaccharide fraction of bioprocessed *Ecklonia cava* extracts (0.09, 0.9, 9 μg/mL) in Tyroid buffer (200 μL; 137 mM NaCl, 2.7 mM KCl, 1.8 mM CaCl_2_, 1.1 mM MgCl_2_, 11.9 mM NaHCO_3_, 0.4 mM NaH_2_PO_4_, 5.6 mM glucose, pH 7.2) were added to each well and incubated for 15 min. To stimulate cells after removal of the extracts by washing with Tyrode buffer, ionophore A23187 (10 μM) was added for 20 min. The supernatant (50 μL) containing released β-hexosaminidase was recovered by centrifugation and then mixed with the same volume of *p*-nitrophenyl-N-acetyl-β-glucosaminide solution (1 mM, pH 5.2) and incubated for 1 h at room temperature. The reaction was terminated by adding sodium carbonate buffer (67 mM, pH 10.2). The absorbance of the supernatant was read at 405 nm using a microplate reader (VersaMax, Molecular Devices Corp., CA, USA).

#### Measurement of the total IgE secretion

Measurement of immunoglobulin E (IgE) production using the B cell line was determined using our published method [13]. To assess changes in IgE production levels, U266.B1 cells were stimulated with 10 μg/mL lipopolysaccharide (LPS), 5 ng/mL human IL-4, and either of the extracts (EC-1, *Ecklonia cava* water extracts (1, 10, 100 μg/mL); EC-2, *Ecklonia cava* 70% ethanol extracts (0.41, 4.1, 41 μg/mL); EC-F-1, bioprocessed *Ecklonia cava* extract (1, 10, 100 μg/mL); EC-F-2, solid-liquid separation fraction of bioprocessed *Ecklonia cava* extracts (0.31, 3.1, 31 μg/mL); EC-F-3, water-soluble fraction of bioprocessed *Ecklonia cava* extracts (0.29, 2.9, 29 μg/mL); EC-F-4, polysaccharide fraction of bioprocessed *Ecklonia cava* extracts (0.09, 0.9, 9 μg/mL) for 72 h. The supernatants were recovered for IgE assay by a commercial kit (Biorbyt, San Francisco, CA, USA) according to the manufacturer’s instructions. Absorbance of the final reaction mixture was read at 450 nm using a microplate reader.

#### Mice

Pathogen-free female Balb/c mice, 6 weeks old, were purchased from Koatech (Gyunggi-do, Korea). The mice were housed in a stainless-steel cage under a 12 h light/dark cycle with a temperature range of 23 °C ± 3 °C and relative humidity of 50 ± 10%. Mice were fed the pelletized normal commercial chow diet (Cat. No. 5 L79, Orient Bio, USA) and tap water ad libitum for 1 week after arrival for acclimation.

#### Antigen sensitization, challenge, and treatment

The protocol for sensitization and inhalational challenge was carried out according to the method of Temelkovski et al. [21] as described in our previous study with slight modification [13].

**Fig. 2:**
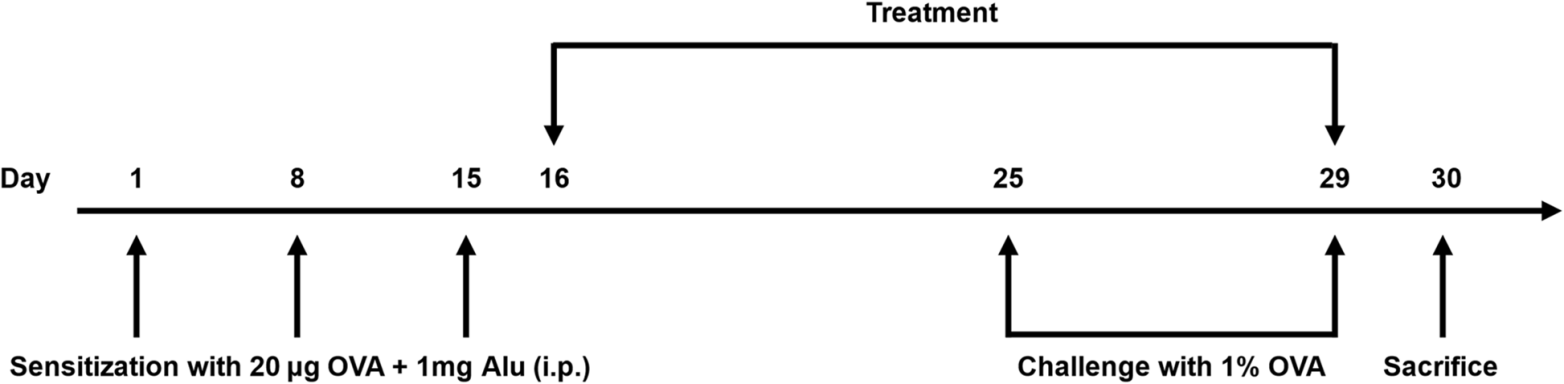
Sensitization scheme of an asthma mouse model

Briefly, acclimatized Balb/c mice were arbitrarily divided into the following groups (*n* = 10), avoiding any intergroup difference in body weight: vehicle, OVA, bioprocessed *Ecklonia cava* and its purified fractions (EC-1, EC-2 – extracts of *E. cava*; EC-F-1 -bioprocessed product; and EC-F-2, EC-F-3, and EC-F-4 -isolated fractions of bioprocessed product). Mice in the OVA sensitization/challenge group were intraperitoneally sensitized with OVA) (20 μg) emulsified with 0.2 mL of 1 mg aluminum hydroxide in pH 7.4 phosphate-buffered saline (PBS). Mice in the vehicle group were also interperitoneally sensitized with PBS (0.2 mL, pH 7.4). Injections were performed three times on days 1, 8, and 15. The sensitized mice were subjected to OVA challenge by placing each mouse individually in a Plexiglas box (43.5 × 27.5 × 31.5 cm). Challenge was continued with repeated exposure to an aerosol of OVA (1%) using an ultrasonic nebulizer (NE-U12, Omron Co., Kyoto, Japan). Challenge was then carried out for 30 min once a day for 5 consecutive days (day 25 to 29). The vehicle-treated group was subjected to PBS exposure. For the treated groups, mice sensitized/challenged with OVA as described were orally administered with either of the extracts for 14 consecutive days (day 16 to 29). PBS was used as vehicle, and all extracts were administered in the diet. All mice were sacrificed by CO_2_ inhalation 24 h after the last (day 30) to assess the asthma inhibitory effect of bioprocessed *Ecklonia cava* and its purified products. The described experimental design is schematically shown in Fig. 2.

#### Collection of bronchoalveolar fluid (BALF) and blood

Collection of BALF and blood was performed using our published method [13]. For the collection of BALF, tracheotomy was performed as follows. The tracheas exposed by cannulating upper tracheas and BALF were carefully collected by twice lavaging with ice-cold PBS (1 mL). Collected lavage fluids were then centrifuged at 2000 *g* for 1 min at 4 °C. The recovered supernatants were set aside and kept at − 70 °C until further analysis. Cell pellets were resuspended in warm RPMI 1640 media, followed by slide preparation by centrifuge (Micro 17R, Hanil Science, Incheon, Republic of Korea) and staining with Wright−Giemsa stain. The slides were observed at a magnification× 40 for differential cell counts by counting a total of 300 cells per slide under light microscopy (CKX41, Olympus, Tokyo, Japan). The total cell number in BALF was also measured by microscopic cell counting using a hemocytometer. Blood was collected by cardiac puncture from the sacrificed mice.

#### ELISA measurement of OVA-specific IgE level in BALF

The OVA-specific IgE release into BALF was measured with a mouse ovalbumin-specific IgE ELISA assay kit (Bio-Rad, Hercules, CA, USA) according to the manufacturer’s instructions. Briefly, appropriately diluted BALF was transferred to 96-well microplate pre-coated with rat anti-mouse IgE monoclonal antibody and incubated for 1 h at room temperature. After washing thorough with diluted wash buffer, horseradish peroxidase (HRP)-conjugated ovalbumin was added to the wells and incubated for an additional 1 h at room temperature. After final wash, the substrate solution was added, and the wells incubated for an additional 30 min in the dark. After the reaction was completed, the absorbance of each well at 450 nm was measured using a microplate reader. OVA-specific IgE concentrations were calculated based on serial dilutions of standard mouse IgE included in the ELISA kit.

#### ELISA measurement of cytokine levels in serum or BALF

Interleukin (IL-2, IL-10, and IL-12) levels in serum, along with IL-4, IL-5, and IL-13 levels in BALF, were measured according to the manufacturer’s instructions for an ELISA kit (R&D Systems, Minneapolis, MN, USA). After experiments according to the manufacturer’s manual, the absorbance of the final mixture was read with a microplate reader at 450 nm. Standard curves for each target substance were created using the standard provided in each ELISA kit.

#### ELISA measurements of OVA-specific IgG subclass levels in serum

Immunoglobulin G (IgG) in mouse serum was measured using an ELISA kit (MyBioSource, San Diego, CA, USA) according to the manufacturer’s protocol. In addition, The IgG1 and IgG2a levels were measured using a published method [13]. Briefly, 96-well plates were pre-coated with 100 μL of OVA solution (5 μg/mL in PBS) overnight at 4 °C. After washing with PBS 3 times, blocking was induced by reacting the plates with 150 μL of 1% bovine serum albumin (BSA) solution for 30 min at room temperature. Serum samples (50-fold dilution for IgG2 and 5000-fold dilutions for IgG1) were then added to the wells and incubated for 2 h at room temperature. HRP-conjugated rat anti-mouse IgG1 and IgG2a (Life Technologies, Carlsbad, CA, USA) was added, and samples were incubated for 2 h at room temperature. The plates were then washed 3 times with PBS-T and TMB (100 μL) substrate was added to each well. After 30 min incubation in the dark, absorbance was read at 450 nm using a microplate reader. Fold increase was calculated based on normal mice for measurements of IgG1 and IgG2a.

#### ELISA measurement of eotaxin, VCAM-1, LTC_4_, and PGD_2_ levels in BALF

Eotaxin and VCAM levels in BALF were measured according to the manufacturer’s instructions for an ELISA kit (Biorbyt, San Francisco, CA, USA). The absorbance of the final mixture was read with a microplate reader at 450 nm. LTC_4_ and PGD_2_ levels in BALF were also determined using another ELISA kit (MyBioSource, San Diego, CA, USA) using manufacturer’s instructions. For both assays, absorbance of the final mixtures at 420 nm was measured using a microplate reader. Standard curves for each target substance were created using the standard provided in each ELISA kit.

#### Histological analysis of the lung

Histological analysis of lung was performed using our published method [13]. The exsanguinated left lung was removed from the chest cavity and fixed with 4% paraformaldehyde in PBS buffer (0.5 M, pH 7.4). Lobes were isolated, dehydrated with ethanol, and embedded in paraffin. The tissues were then cut to a thickness of 4 μm and mounted onto glass slides. To observe inflammatory cell infiltration, the sections were dewaxed using xylene and ethanol, stained with hematoxylin and eosin (H&E), and examined by light microscopy (CKX41, Olympus, Tokyo, Japan).

#### Statistical analysis

Results are expressed as the mean ± SD of three independent repetitions. Significant differences between means were determined by the ANOVA test using the Statistical Analysis Software package SAS (Cary, NC, USA). *p* < 0.05 is regarded as significant.

## Results

### Analysis of dieckol in bioprocessed *Ecklonia cava* and its purified products

The solid-liquid separation, water-soluble, and polysaccharide fractions obtained by sequential purification of bioprocessed *Ecklonia cava* were freeze-dried. The following amounts of dieckol, a standard bioactive component of *Ecklonia cava*, were recovered in the lyophilized fractions: bioprocessed *Ecklonia cava*, 10.9 mg/g; solid-liquid separation fraction, 4.3 mg/g; water-soluble fraction, 3.9 mg/g; and polysaccharide fraction (below the limit of quantitation), 2 mg/g (Fig. 1).

### Analysis of saccharides in the polysaccharide fraction of bioprocessed *Ecklonia cava*

**Fig. 3:**
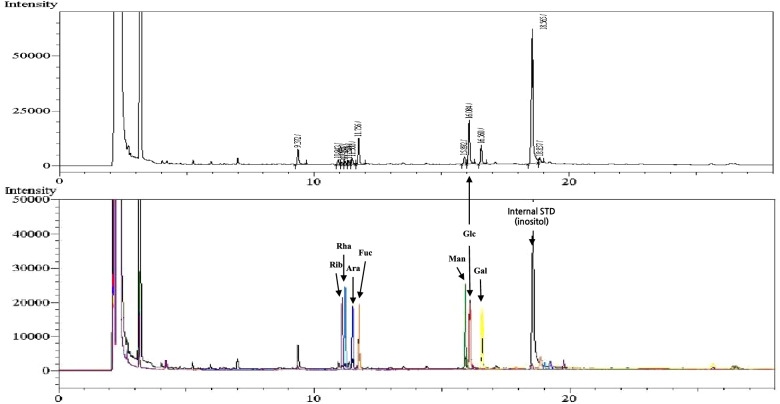
Chromatogram of monosaccharides in a polysaccharide fraction isolated from bioprocessed *Ecklonia cava*

Eleven monosaccharide standards were used to characterize the individual monosaccharides in the polysaccharide fraction by the following method. The polysaccharide fraction was acid hydrolyzed, derivatized, and then analyzed using GC-FID. Table 1 shows that 7 identified peaks have the same retention times as the monosaccharide standards. Glucose was present as the highest amount in the mixture. In addition, there are numerous unknown peaks (Fig. 3). These fractions require further characterization.

**Table 1:** Gas chromatography (GC) data of monosaccharides as acetylated aldononitriles

	Monosaccharide name	Retention time (min)	Peak area	Portion (%)
1	Ribose	11.1	4071	0.76
2	Rhamnose	11.2	6280	1.17
3	Arabinose	11.5	10,282	1.91
4	Fucose	11.8	44,058	8.19
5	Mannose	15.9	12,888	2.40
6	Glucose	16.1	71,770	13.35
7	Galactose	16.6	31,996	5.95
Total AUC			537,740	41.92^a^

^a^ Excludes unknown peak area., Additional purification of the polysaccharide fraction is required

### Inhibition of the activation of mast cells by bioprocessed *Ecklonia cava* and isolated fractions

Since most allergic diseases are associated with hypersensitivity reactions caused by exposure to a specific antigen, we investigated the modulating effect of the bioprocessed *Ecklonia* cava and its purified products on the allergic inflammatory response. We observed that degranulation was induced and the amount of β-hexosaminidase in the supernatant was increased in A23187-treated control RBL-2H3 cells. By contrast degranulation was inhibited by bioprocessed *Ecklonia cava* in RBL-2H3 cells in a dose-dependent manner following treatments without apparent toxicity to cells (data not shown). In addition, treatment with EC-F-1 (100 μg/mL) resulted in 23% inhibition. The activities of purified fractions (EC-F-2, EC-F-3, and EC-F-4) were gradually decreased as the purification progressed. There was almost no activity in the polysaccharide fraction (Table 2). These results suggest that the effect of inhibiting the degranulation of mast cells is due to phlorotannin, an active component present in the EC-1 fraction, and as the purification progresses, the amount of active phlorotannin is reduced.

**Table 2:** Inhibitory effect of bioprocessed *Ecklonia cava* and its purified products on β-hexosaminidase releases stimulated by A23187 in RBL-2H3 cells

		β-hexosaminidase release (%)
**Vehicle**		0 ± 10^e^
**Positive control (10 μM calcium ionophore A23187)**		100 ± 15^a^
**EC-1**	1 μg/mL	93 ± 12^ab^
	10 μg/mL	78.1 ± 4.0^bc^
	100 μg/mL	66 ± 10^d^
**EC-2**	0.41 μg/mL	84.4 ± 4.3^b^
	4.1 μg/mL	73.0 ± 9.5^c^
	41 μg/mL	62.5 ± 7.1^d^
**EC-F-1**	1 μg/mL	91.5 ± 9.7^ab^
	10 μg/mL	81.3 ± 3.5^b^
	100 μg/mL	76.7 ± 2.3^c^
**EC-F-2**	0.31 μg/mL	95.5 ± 6.0^a^
	3.1 μg/mL	87.5 ± 3.5^ab^
	31 μg/mL	83.8 ± 4.2^b^
**EC-F-3**	0.29 μg/mL	98.6 ± 7.7^a^
	2.9 μg/mL	94.6 ± 3.1^a^
	29 μg/mL	90 ± 12^ab^
**EC-F-4**	0.09 μg/mL	102 ± 12^a^
	0.9 μg/mL	98.6 ± 3.0^a^
	9 μg/mL	94.0 ± 6.8^a^

Data are shown as the mean ± SD of three independent repetitions. PBS was used as vehicle. Values in each column with the same superscript letters a-e are not significantly different between groups at *p* < 0.05

### Effects of bioprocessed *Ecklonia cava* and isolated fractions on total IgE production in U266.B1 human multiple myeloma cells

U266.B1 cells, LPS (10 μg/mL), and IL-4 (5 ng/mL) were treated with the extracts at three concentrations and the inhibition of IgE production was determined. The results show that in the LPS and IL-4 treatment group IgE levels increased about 62-fold (489 ± 52 ng/mL) compared to the normal group (7.9 ± 1.6 ng/mL). By contrast, IgE production was dose-dependently inhibited, and toxicity to cells was not observed (data not shown) as demonstrated by the following results. Treatment with EC-1 (100 μg/mL) and EC-2 (41 μg/mL) caused a 12% decrease in IgE production. Treatment with EC-F-1 (100 μg/mL) and purified fractions (EC-F-2, EC-F-3, and EC-F-4) resulted in inhibitory effects of 55, 58, 53, and 61%, respectively. Table 3 also shows that these inhibitory effects were all higher than the effect with EC-1. Because the differences in the inhibition of IgE formation among the extracts for each sequentially purified fraction of EC-F-1 was not significant, the IgE production inhibitory effect of the extract can be defined by the effect of the polysaccharide generated through the bioconversion process.

**Table 3:** Effects of bioprocessed *Ecklonia cava* and its purified products on total IgE production in U266.B1 human multiple myeloma cells

		IgE concentration (ng/mL)
**Vehicle**		7.9 ± 1.6^e^
**Positive control: (10** μg**/mL LPS + 5 ng/mL IL-4)**		489 ± 52^a^
**EC-1**	1 μg/mL	473 ± 44^a^
	10 μg/mL	464 ± 40^a^
	100 μg/mL	442 ± 52^ab^
**EC-2**	0.41 μg/mL	466 ± 42^a^
	4.1 μg/mL	448 ± 38^ab^
	41 μg/mL	433 ± 41^ab^
**EC-F-1**	1 μg/mL	419 ± 36^b^
	10 μg/mL	318 ± 33^c^
	100 μg/mL	224 ± 29^d^
**EC-F-2**	0.31 μg/mL	408 ± 32^b^
	3.1 μg/mL	316 ± 28^c^
	31 μg/mL	209 ± 19^d^
**EC-F-3**	0.29 μg/mL	431 ± 36^ab^
	2.9 μg/mL	356 ± 31^bc^
	29 μg/mL	232 ± 25^d^
**EC-F-4**	0.09 μg/mL	394 ± 42^b^
	0.9 μg/mL	308 ± 28^c^
	9 μg/mL	194 ± 23^d^

Data are shown as the mean ± SD of three independent repetitions. PBS was used as vehicle. Values in each column with the same superscript letters a-e are not significantly different between groups at *p* < 0.05

### Inhibition of TSLP production in bronchoalveolar lavage (BALF)

**Fig. 4:**
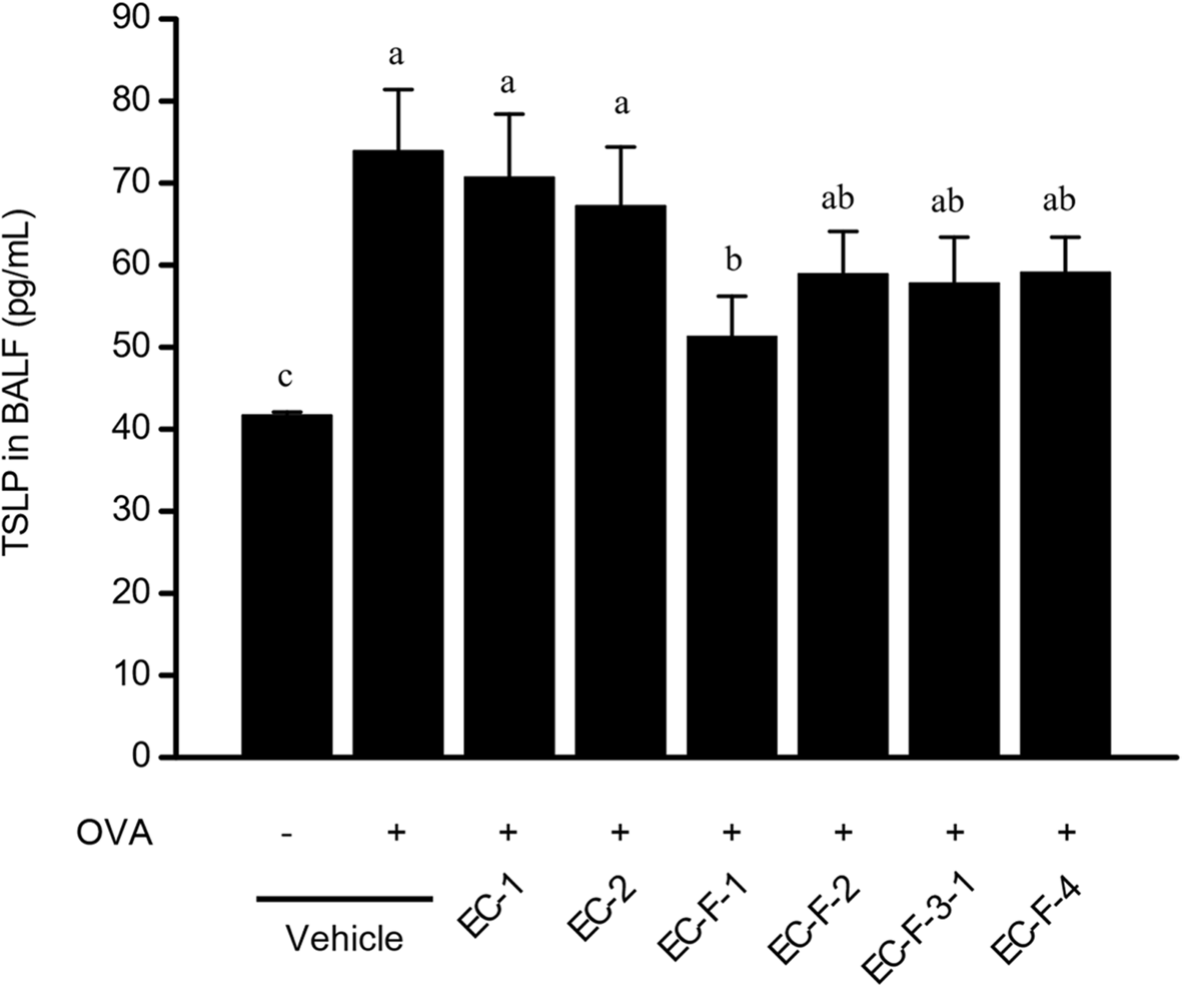
Decreased levels of thymic stromal lymphopoietin (TSLP) level in bronchoalveolar lavage fluid (BALF) from OVA-sensitized/challenged allergic model mice orally administered with bioprocessed *Ecklonia cava* and its purified products. BALF was collected from *Ecklonia cava* extracts-administered sensitized/challenged Balb/c mice by lavaging tracheas with PBS. After centrifugation, supernatant was recovered from each mouse group or quantitation of TSLP level in BALF using the ELISA kit. Representation: vehicle (−), negative control not sensitized/challenged with OVA; vehicle (+), OVA-sensitized/challenged positive control; EC-1, *Ecklonia cava* water extracts (40 mg/kg body weight); EC-2, *Ecklonia cava* 70% ethanol extracts (20 mg/kg body weight); EC-F-1, bioprocessed *Ecklonia cava* extract (40 mg/kg body weight); EC-F-2, Solid-liquid separation fraction of bioprocessed *Ecklonia cava* (12 mg/kg body weight); EC-F-3, Water-soluble fraction of bioprocessed *Ecklonia cava* (11 mg/kg body weight); EC-F-4, Polysaccharide fraction of bioprocessed *Ecklonia cava* (3 mg/kg body weight), mouse groups dietary administered with all extracts respectively. Data are expressed as mean ± SD (*n* = 10). Bars sharing a common letter are not significantly different between groups at *p* < 0.05

The inhibitory effect on TSLP production in bronchoalveolar lavage (BALF) was determined by the enzyme-linked immuno-sorbent assay (ELISA) assay. The results show that the amount of TSLP in the BALF by OVA stimulation was increased 1.7 times in the asthma induction group (73.9 ± 7.5 pg/mL) compared to the normal group (41.7 ± 0.4 pg/mL). For the dietary group treated with the bioprocessed *Ecklonia cava* and isolated products mentioned above, Fig. 4 shows that the inhibitory effects were 10, 21, 70, 47, 50, and 46%, respectively. The EC-F-1 diet group showed the best effect (70%), and, as in the case of the fractions, the efficacy was partially reduced owing to the loss of active ingredients present in the *Ecklonia cava*, such as phlorotannin. However, most of the treatment groups showed a high inhibitory effect, and there was little difference between the isolated fractions.

### Inhibition of OVA-specific IgE production in BALF

**Fig. 5:**
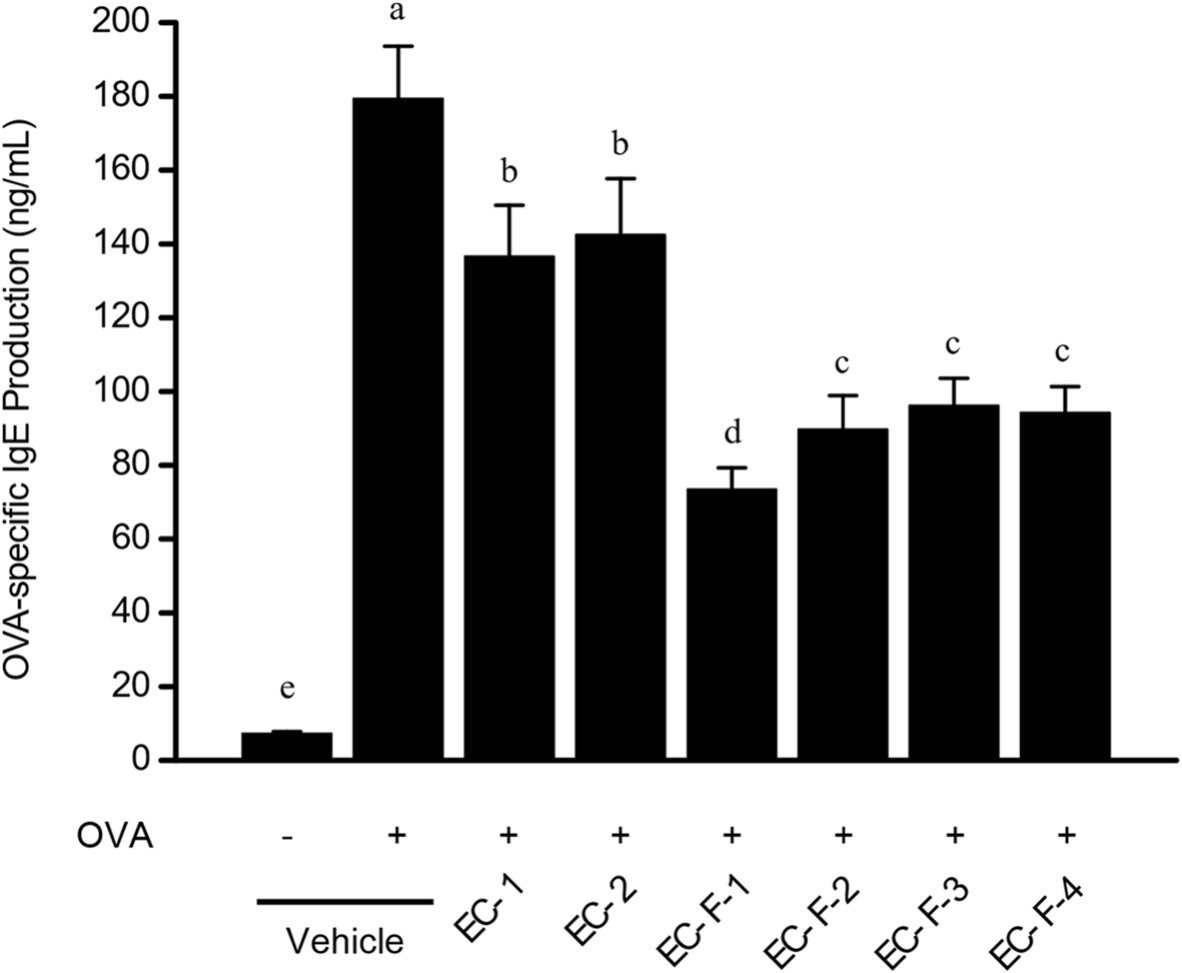
OVA-specific IgE level in bronchoalveolar lavage fluid (BALF) from OVA-sensitized/challenged allergic model mice orally administered with bioprocessed *Ecklonia cava* and its purified products. BALF was collected from *Ecklonia cava* extracts-administered sensitized/challenged Balb/c mice by lavaging tracheas with PBS. After centrifugation, supernatant was recovered from each mouse group or quantitation of OVA-specific IgE level in BALF using the ELISA kit. Data are expressed as mean ± SD (*n* = 10). Bars sharing a common letter are not significantly different between groups at *p* < 0.05

The fractions were analyzed by ELISA to investigate if OVA stimulation induces IgE-mediated asthma, and if the inhibitory effect on OVA-specific IgE was produced in BALF of OVA-induced asthma mice after feeding bioprocessed *Ecklonia cava*. The results show that the amount of OVA-specific IgE in the BALF of the asthma-inducing group (179 ± 14 ng/mL) increased about 25 times compared to the normal group (7.3 ± 0.5 ng/mL). The daily administration of bioprocessed *Ecklonia* cava and its purified products resulted in inhibitions of 25, 21, 62, 52, 48, and 49%, respectively (Fig. 5). As with the above-mentioned results, the greatest effect was observed with EC-F-1.

### Inhibition of the production of OVA-specific immunoglobulin isotypes (IgG, IgG1, IgG2a) in serum

**Fig. 6:**
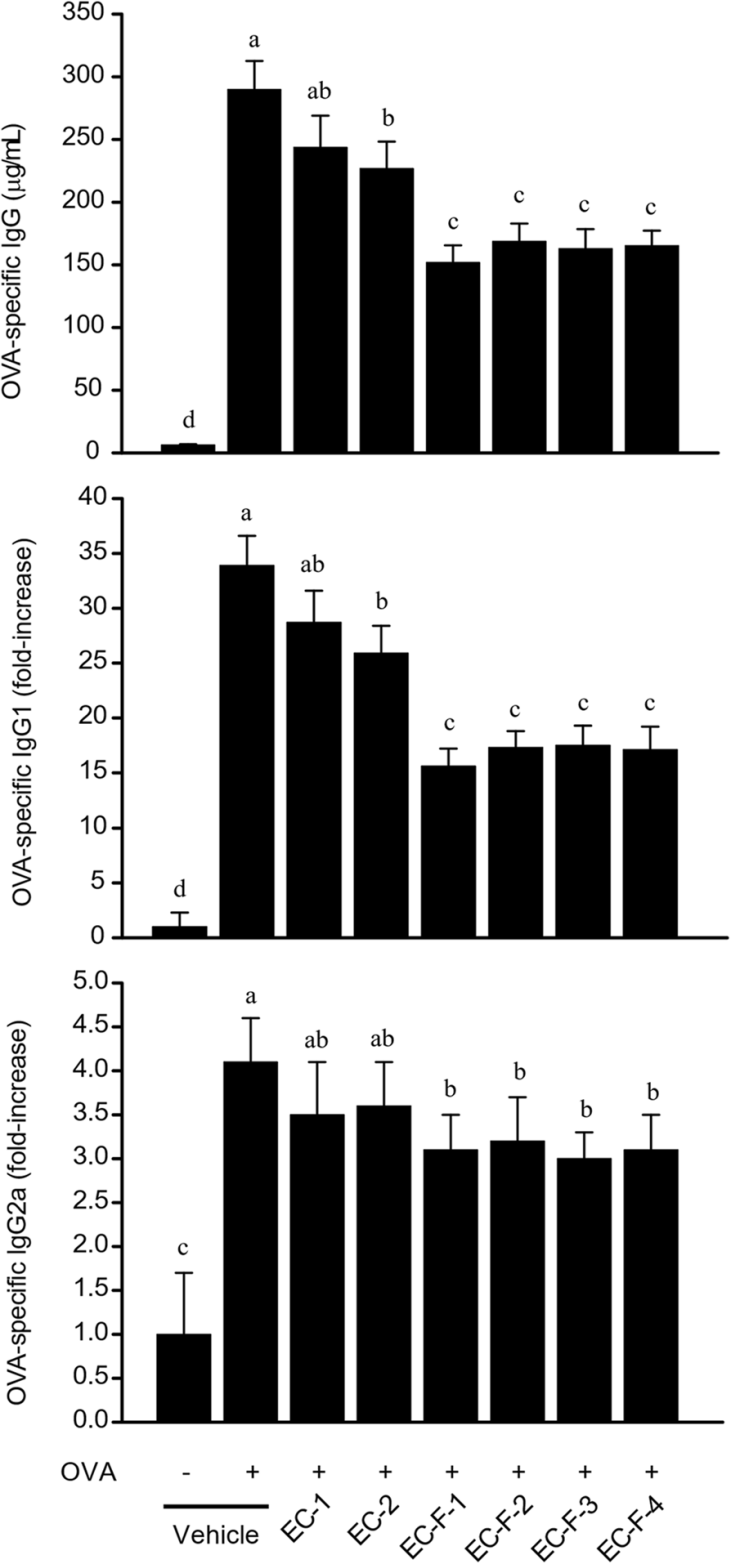
OVA-specific IgG, IgG1, and IgG2a levels in serum from OVA-sensitized/challenged asthma model mice administered with bioprocessed *Ecklonia cava* and its purified products. The sensitized/challenged Balb/c mice were orally administered with *Ecklonia cava* extracts and were sacrificed to bleed by cardiac puncture. Serum was collected after blood clotting reaction, and the resultant supernatant was quantitatively assayed for IgG, IgG1, and IgG2a levels of each mouse group using the ELISA method. Data are expressed as mean ± SD (n = 10). Bars sharing a common letter are not significantly different between groups at *p* < 0.05

Feeding bioprocessed *Ecklonia cava* and isolated fractions to OVA-induced asthma mice affected the production of OVA-specific immunoglobulin isotypes (OVA-specific IgG, IgG1, and IgG2a). The total IgG, IgG1, and IgG2a resulting from the induction of asthma was an approximately 46-, 34-, and 25-fold increase, respectively, compared with normal mice. Figure 6 shows that, in contrast, total IgG in the serum of the bioprocessed *Ecklonia cava* and its purified products diet group showed inhibitory effects of 16, 22, 49, 43, 45, and 44%, respectively, and for IgG1 the values are 16, 24, 56, 50, 50, and 51% respectively. The corresponding inhibitions for IgG2a are 19, 16, 32, 29, 35, and 32%, respectively. The greatest effect was observed with EC-F-1, the unmodified bioprocessed product. The data show that the bioprocessed *Ecklonia cava* and its purified products used in this study had a much greater inhibitory effect on IgG1 generated by the Th2 immune response compared to the inhibitory effect on IgG2a generated by the Th1 immune response.

### Effects of bioprocessed *Ecklonia cava* and isolated fractions on the secretion of Th1, Th2, and Treg cytokines

The following observations demonstrate changes in the Th1, Th2, and Treg immune responses induced by the bioprocessed *Ecklonia cava* and its purified products in asthma-induced mice.

IL-4, IL-5, and IL-13 in BALF were measured to be about 10 times greater following the induction of asthma by OVA as compared to normal mice. The administration of EC-1 (40 mg/kg) inhibited IL-4, IL-5, and IL-13 production by 9, 14, and 19%, respectively. The corresponding inhibition values with EC-2 (20 mg/kg) are 13, 21, and 15%, respectively, and with EC-F-1 (40 mg/kg) are 39, 41, and 50%, respectively. The administration of purified fractions of EC-F-1 (EC-F-2, EC-F-3, and EC-F-4) all resulted in a sequentially reduced inhibition of IL-4, IL-5, and IL-13 as the purification progressed.

The observed reductions in IL-2 and IL-12 in the serum by ~ 40–60% compared to normal mice confirm that asthma was induced by OVA. The administration of EC-1 (40 mg/kg) resulted in 13 and 37% recovery of IL-2 and IL-12 levels, respectively; the corresponding values for EC-2 (20 mg/kg) are 10 and 31% recovery, respectively. With EC-F-1 (40 mg/kg), however, recovery levels were much higher at 51 and 64%, respectively. Table 4 also shows that the recovery of IL-2 and IL-12 levels resulting from administering the purified fractions (EC-F-2, EC-F-3, and EC-F-4) decreased sequentially as the purification progressed.

**Table 4:** Effects of bioprocessed *Ecklonia cava* and its purified products on Th1, Th2, and Treg cytokine release into serum and BALF from OVA-sensitized/challenged mice

	Cytokine Production (ng/mL)					
	in BALF			in Serum		
	IL-4	IL-5	IL-13	IL-2	IL-12	IL-10
**Vehicle**	8.3 ± 0.7^e^	11.7 ± 1.3^e^	11.4 ± 1.4^e^	18.3 ± 1.7^a^	222 ± 23^a^	88.4 ± 7.9^a^
**OVA only**	82.7 ± 5.8^a^	133 ± 13^a^	131 ± 15^a^	7.9 ± 0.8^e^	132 ± 12^e^	34.8 ± 4.2^f^
**EC-1**	75.9 ± 6.2^ab^	115.9 ± 9.3^ab^	107.8 ± 9.7^b^	9.2 ± 1.4^d^	165 ± 15^d^	42.7 ± 3.7^e^
**EC-2**	72.7 ± 5.4^b^	108 ± 11^b^	113 ± 14^ab^	8.9 ± 0.9^d^	159 ± 14^d^	40.7 ± 3.7^e^
**EC-F-1**	53.6 ± 4.9^d^	83.6 ± 7.2^d^	71.2 ± 8.3^d^	13.2 ± 1.7^b^	190 ± 16^b^	66.9 ± 5.8^b^
**EC-F-2**	59.2 ± 5.2^cd^	88.9 ± 6.9^cd^	78.6 ± 5.6^cd^	12.6 ± 0.8^b^	176 ± 15^bc^	58.2 ± 5.2^c^
**EC-F-3**	63.9 ± 5.9^c^	85.3 ± 7.3^d^	86.3 ± 4.9^c^	10.7 ± 1.3^c^	172 ± 19^c^	49.6 ± 5.5^d^
**EC-F-4**	66.2 ± 7.2^bc^	91.2 ± 7.4^c^	81.2 ± 9.6^c^	11.5 ± 1.7^bc^	181 ± 17^b^	52.2 ± 6.2^d^

Data are shown as the mean ± SD of three independent repetitions. PBS was used as vehicle. Values in each column with the same superscript letters a-e are not significantly different between groups at *p* < 0.05

Similarly, an evaluation of the change in IL-10 expressed through CD4+ regulatory T cells in the serum shows a 60% reduction in asthma induced by OVA compared to that in normal mice. The data also show that the IL-10 decrease induced by OVA was restored following treatment with bioprocessed *Ecklonia cava* and its purified products by 15, 60, 44, 28, 32, and 11%, respectively.

### The effect of bioprocessed *Ecklonia cava* and isolated fractions on the reduction of immune cells in BALF

**Fig. 7:**
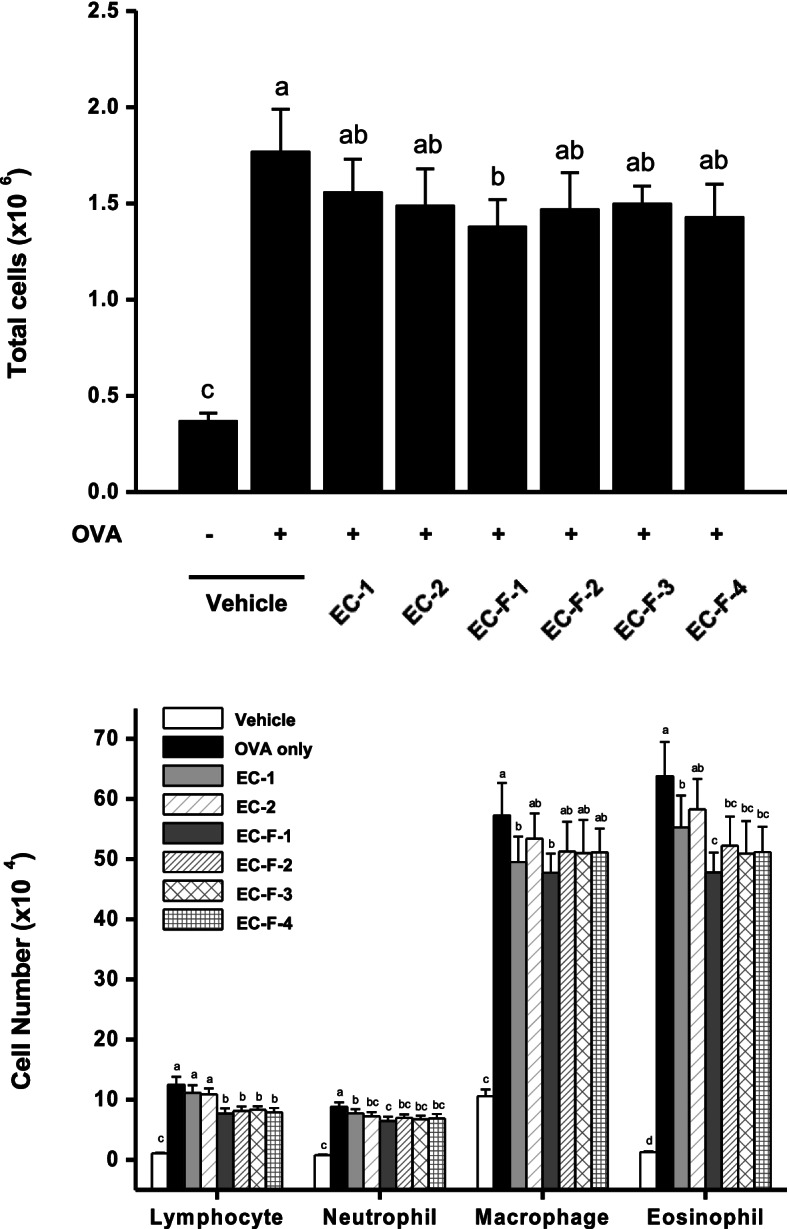
Inhibition of leukocyte infiltration into BALF by bioprocessed *Ecklonia cava* and its purified products administrations in OVA-sensitized/challenged mice. **A** Total leukocyte infiltration changes by bioprocessed *Ecklonia cava* and its purified products administration. **B** Lymphocyte, neutrophil, macrophage, and eosinophil infiltration profile changes by bioprocessed *Ecklonia cava* and its purified products. BALF was centrifuged to obtain cell pellets, which were resuspended in PBS, followed by centrifuging onto slide glass and subsequent staining with Wright−Giemsa staining. The slides were microscopically observed (magnification, × 40) for differential cell count by counting a total of 300 cells per slide. Total cell number in BALF was measured by cell counting using a hemocytometer. Data are expressed as mean ± SD (*n* = 10). Bars sharing a common letter are not significantly different between groups at *p* < 0.05

Immune cell population changes in BALF were investigated in mice with asthma induced by OVA stimulation. The results show that total immune cells in the BALF increased more than 5 times in the asthma-induced group (1.77 ± 0.22 × 10^6^ cells) compared to the normal group (0.37 ± 0.04 × 10^6^ cells). The daily administration of bioprocessed *Ecklonia cava* and purified products resulted in inhibitions of 15, 20, 28, 21, 19, and 24%, respectively, indicating a higher inhibition than by EC-1 used as control. In addition, the infiltration of lymphocytes, neutrophils, macrophages, and eosinophils into the BALF increased by OVA stimulation (about 12, 11, 5 times, and 49 times, respectively), confirming that the infiltrations were inhibited by administration of bioprocessed *Ecklonia cav*a and fractions (Fig. 7).

### The effects of bioprocessed *Ecklonia cava* and isolated fractions on chemoattractants and eicosanoids levels in the BALF

Chemoattractants and eicosanoids, such as eotaxin, VCAM-1, LTC_4_ and PGD_2,_ levels were also determined as biomarkers of airway inflammation. The results shows that eotaxin, VCAM-1, LTC_4_, and PGD_2_ levels were increased by the induction of asthma compared with those in normal mice (about 5.8-, 11.3-, 3.4-, and 2.9-fold, respectively). In contrast, eotaxin content in the BALF after the dietary administration of bioprocessed *Ecklonia cava* and fractions showed inhibitory effects of 30, 27, 61, 55, 54, and 54%, respectively. The corresponding inhibitory values for VCAM-1 are 30, 23, 64, 61, 59, and 60%, respectively. For LTC_4_ in the BALF, the bioprocessed *Ecklonia cava* and fractions showed inhibitory effects of 32, 27, 59, 53, 53, and 55%, respectively. For PGD_2_, the corresponding reductions are 31, 25, 59, 55, 53, and 51%, respectively. As shown above with the previous results, the greatest inhibitory effect was observed with the bioprocessed product EC-F-1 (Table 5).

**Table 5:** Effects of bioprocessed *Ecklonia cava* and its purified products on chemoattractant and eicosanoid releases into BALF from OVA-sensitized/challenged mice

	Chemoattractants (pg/mL)		Eicosanoids (ng/mL)	
	eotaxin	VCAM-1	LTC_**4**_	PGD_**2**_
**Vehicle**	21.8 ± 2.2^e^	2.5 ± 0.2^d^	30.7 ± 2.7^d^	28.5 ± 1.7^d^
**OVA only**	127 ± 12^a^	28.3 ± 1.9^a^	105.7 ± 8.3^a^	82.4 ± 6.3^a^
**EC-1**	95.7 ± 5.6 ^b^	20.6 ± 1.7^b^	81.9 ± 8.2^b^	65.6 ± 4.8^b^
**EC-2**	98.2 ± 7.2^b^	22.4 ± 1.4^b^	85.6 ± 8.3^b^	69.1 ± 5.2^b^
**EC-F-1**	62.5 ± 5.4^d^	11.8 ± 0.8^c^	61.4 ± 5.7^c^	50.6 ± 4.2^c^
**EC-F-2**	68.9 ± 6.3^c^	12.5 ± 1.2^c^	66.3 ± 4.9^c^	52.8 ± 3.7^c^
**EC-F-3**	70.3 ± 5.8^c^	13.1 ± 1.1^c^	65.7 ± 4.2^c^	53.6 ± 4.9^c^
**EC-F-4**	70.1 ± 6.9^c^	12.8 ± 0.9^c^	64.8 ± 6.2^c^	55.1 ± 5.1^c^

Data are shown as the mean ± SD of three independent repetitions. PBS was used as vehicle. Values in each column with the same superscript letters a-e are not significantly different between groups at *p* < 0.05

### Inhibitory effect on inflammation in lung tissue

**Fig. 8:**
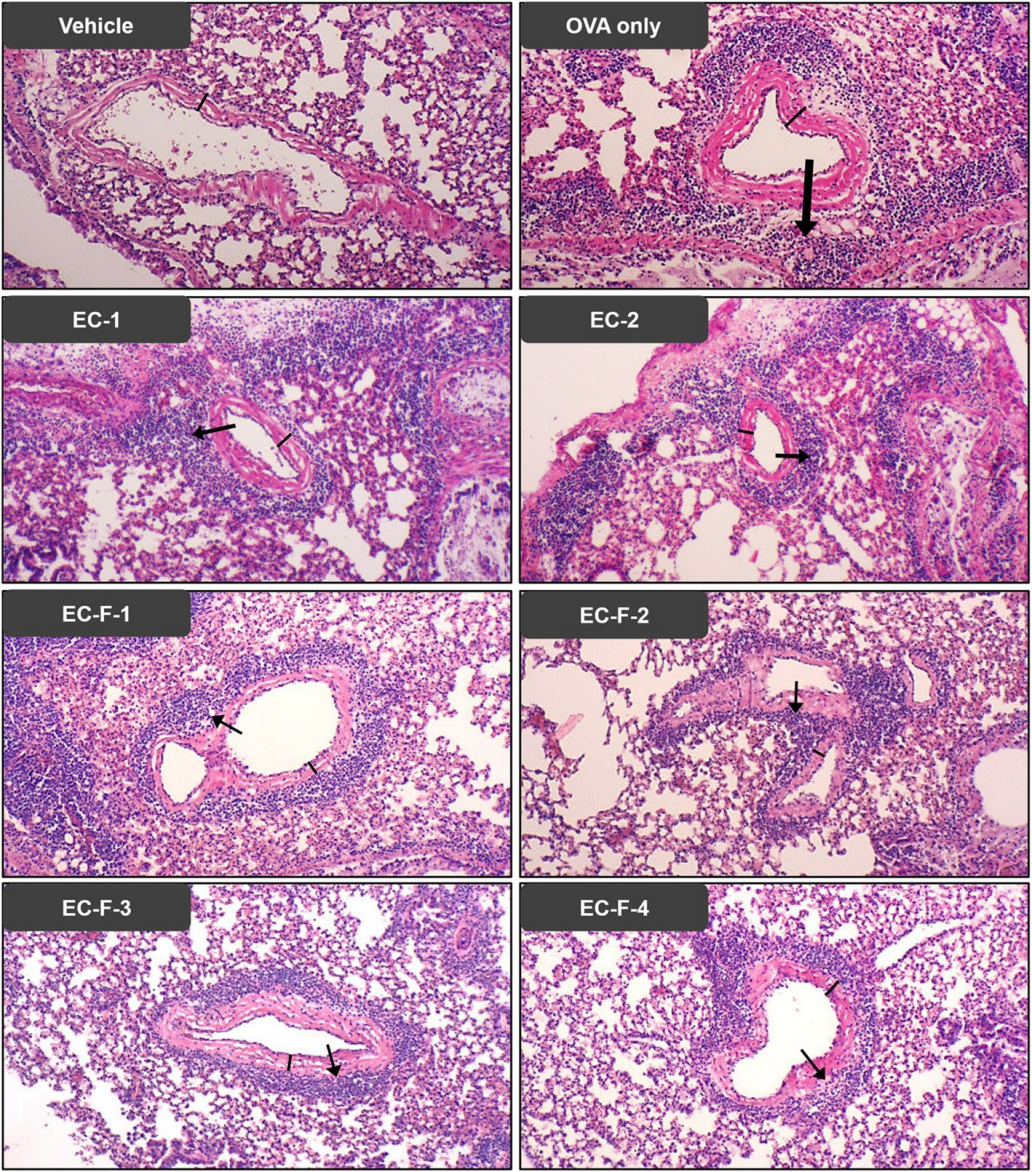
Morphological changes in the lung tissues of OVA-sensitized/challenged allergic mice orally administered with bioprocessed *Ecklonia cava* and its purified products. Lung tissues from sensitized/challenged Balb/c mice were fixed with 10% (v/v) paraformaldehyde. The fixed tissues were sectioned to 4 μm, followed by staining with hematoxylin and eosin (H&E) and light microscopy (magnification, × 100. The black arrows indicate the aggregation of inflammatory cells, and the black vertical line indicates the airway wall thickness. Data are expressed as mean ± SD (*n* = 10). Bars not sharing a common letter are not significantly different between groups at *p* < 0.05. Arrows indicate the level of tracheal edema that resulted from inflammatory cell infiltration in lung tissue from each mouse group. Figures represent the average of at least three individual repetitions

Examination of the lung tissue slides stained with hematoxylin and eosin revealed the thickening of the airway wall and the contraction and infiltration of bronchial and blood vessels and perialveolar inflammatory cells. These results show increased bronchial wall thickening and inflammatory cell infiltration in lung tissue in the OVA-induced asthma group compared to the normal mouse group. In contrast, administration of EC-1 (40 mg/kg), EC-2 (20 mg/kg), EC-F-1 (40 mg/kg), EC-F-2 (12 mg/kg), EC-F-3 (11 mg/kg), and EC-F-4 (3 mg/kg) alleviated suppressed infiltration of immune cells and inflammation of lung tissue, confirming the protective effect on lung tissue (Fig. 8). These data show that the inhibition of bronchial wall thickening and contraction was excellent; it resulted in recovery to a level similar to that of the normal group that was administered dietary EC-F-1.

## Discussion

The results of the present study show that the oral administration of new food product prepared by bioprocessing (fermenting) the commercial edible brown algae *Ecklonia cava* with mushroom mycelia ameliorated adverse effects associated with allergic asthma in the OVA-sensitized challenged mice. Specifically, elevated Th1 and other cytokine production was present at near normal levels. In addition, the expression of inflammatory mediators was reduced owing to the apparent reduction in gene expression at the transcription level. The elevated immunoglobulin subclass production directly related to Th2 immune reaction was also reduced, suggesting that the formulation modulates the Th1/Th2 balance by suppressing the Th2 immune response. Treg IL-10 cytokine production was elevated to near normal levels, and the production of chemoattractant biomarkers involved in the inflammation of airways of asthma mice were inhibited. The bioprocessed product also reduced the number of lymphocytes in bronchoalveolar lavage fluid, and had potent suppressive effects on lung airway inflammation, protecting the morphology of lung tissues against inflammatory cell infiltration. These results, and additional parallel studies on the inhibition of asthma in the rat basophilic leukemia mast cell line RBL-2H3, suggest that the OVA-induced suppression of IL-10 production to near normal levels seem to be a critical molecular event in the protection against allergic asthma. In particular, considering that IL-10 has a direct regulatory effect on Th2-mediated allergic airway inflammation [22], the asthma inhibitory effect of bioprocessed *Ecklonia cava* and its purified fractions effectively inhibit Th2-mediated inflammatory responses through activation of Th1 and Treg immune responses.

Additional analytical and chemical studies show that the unmodified *E. cava* algae and several fractions isolated by preparative chromatography of the bioprocessed product also inhibited asthma-associated biomarkers following oral dietary administration to asthmatic mice. We do not know the exact composition, except that all fractions contained the known bioactive compound dieckol, and that one of the fractions might be a polysaccharide based on the observed content of eight monosaccharides. The extent of quantitative inhibitions by *E. cava* and of the fractions were, however, lower than observed with the bioprocessed substance, suggesting that the bioprocessed formulation, not the isolated fractions, merits further clinical human study to confirm the excellent anti-asthma properties observed in cells and mice.

The current study represents our second anti-asthma study. As noted in the introduction, the first anti-asthma study reported by Kim, Lee, Nam and Friedman [13] showed that elm tree (*Ulmus parvifolia*) bark bioprocessed with mycelia of shiitake (*Lentinula edodes*) mushroom mycelia protected mice against asthma by a mechanism that seems to be similar to that described in the present study for a different bioprocessed product. In additional previous studies, we describe the health-promoting properties of several other food formulations. These include: (a) a polysaccharide isolated from the liquid culture of *Lentinula edodes* (shiitake) mushroom mycelia containing black rice bran that protected mice against endotoxemia [14] and salmonellosis [23]; (c) turmeric bioprocessed with *Lentinula edodes* (shiitake) mushroom mycelia that was also effective against salmonellosis in mice [24]; (d) a shiitake (*Lentinula edodes*) mushroom mycelia and rice bran formulation that protected mice against *Salmonella enterica* serovar Typhimurium strain SL1344 in macrophage cells and in mice [25]; (e) a bioprocessed black rice bran glutathione-enriched yeast extract that protected rats and mice against alcohol-induced hangovers [26]; and (f) anti-allergic and anti-inflammatory rice formulations [27–30].

The high bioactivity of the bioprocessed combinations against foodborne pathogens and hangover biomarkers in rodents are most likely the result of the formation of new bioactive compounds during the fermentation process, an aspect that merits further study. Moreover, although asthma does not seem to increase risk of COVID-19 infection, it would also be of interest to determine if the bioprocessed *E. cava* food formulation that protected the lungs of mice against inflammatory allergic asthma might also help ameliorate the acute respiratory syndrome associated with the viral infection [31–33] as well as allergic manifestations associated with foods [34–38] and drugs [39].

## Conclusions

The in vitro cell and in vivo mouse assays demonstrate the potential value of the bioprocessed formulation as an anti-inflammatory and anti-allergic combination of natural compounds, and possibly also as a therapeutic agent for the treatment and prevention of allergic diseases in humans such as hay fever and asthma. Because edible *Ecklonia cava* extracts seem to be safe for consumption, as indicated by the described human toxicity and metabolism assays, and the algae are widely consumed in Asian countries as a natural traditional human medicinal product and are also commercially available in the United States, it is likely that the bioprocessed formulation might be safe for allergic asthmatic patients. The described anti-allergic asthma properties in cells and in mice need, however, to be confirmed by clinical studies with human patients, including asthmatic children, to determine the value of the bioprocessed formulation to prevent and/or treat allergic asthma.

In summary, edible algae bioprocessed with shiitake mushroom mycelia inhibits mast cell degranulation and IgE production in vitro and the pro-inflammatory effects seen in an OVA-induced asthma model in vivo. This novel food product may have useful therapeutic applications. In vivo studies should include an assessment of the ratio of effective to toxic doses.

## Data Availability

The original source of method description is acknowledged and cited in each case. The datasets used and/or analyzed during the current study and supplementary material are available from the following author on reasonable request: Prof. Sung Phil Kim; E-mails: spkim@strbiotech.co.kr; ksp1108@ajou.ac.kr.

## Abbreviations

ANOVA: Analysis of Variance
AUC: Area Under Curve
BALF: Bronchoalveolar Lavage Fluid
BMI: Basal Metabolic Index
BSA: Bovine Serum Albumin
DMEM: Dulbecco’s Modified Eagle’s Medium
ELISA: Enzyme Linked Immunosorbent Assay
FBS: Fetal Bovine Serum
GC-FID: Gas Chromatography-Flame Ionization Detector
HGF-1: Human Gingival Regulatory Fibroblast-1
HPLC: High Performance Liquid Chromatography
HRP: Horse Radish Peroxidase
IgG-1: Immunoglobulin-1
IL-1: Interleukin-1
LPS: Lipopolysaccharide
LTC_4_: Leukotriene C4
OVA: Chicken Egg Ovalbumin
PBS: Phosphate-Buffered Saline
PDA: Potato Dextrose Agar
PGD_2_: Prostaglandin D2
ω-3 PUFAs: ω-3 Polyunsaturated Fatty Acids
RT-PCR: Reverse Transcription-Polymerase Chain Reaction
Th1/Th2: T helper 1/T helper 2
TMB: Tetramethylbenzidine
TNF-alpha: Tumor Necrosis Factor-Alpha
Treg: T Cell Regulatory Cytokine
TSLP: Thymic Stromal Lymphopoietin
VCAM-1: Vascular Cell Adhesion Molecule-1
